# Practical Aspects and Mechanism of Asymmetric Hydrogenation with Chiral Half-Sandwich Complexes

**DOI:** 10.3390/molecules18066804

**Published:** 2013-06-10

**Authors:** Jiří Václavík, Petr Šot, Beáta Vilhanová, Jan Pecháček, Marek Kuzma, Petr Kačer

**Affiliations:** 1 Department of Organic Technology, Institute of Chemical Technology, Technická 5, CZ-166 28 Prague, Czech Republic; 2 Laboratory of Molecular Structure Characterization, Institute of Microbiology, *v.v.i.*, Academy of Sciences of the Czech Republic, Vídeňská 1083, CZ-142 20 Prague, Czech Republic

**Keywords:** asymmetric hydrogenation, ruthenium, reaction conditions, mechanism, imine

## Abstract

This review is oriented toward the asymmetric transfer hydrogenation (ATH) of imines regarding mostly fundamental, yet important topics from the practical point of view. Development of analytical methods for the monitoring of ATH (*i.e.*, kinetics and stereoselectivity) belongs to those topics, as well as studies on the influence of reaction conditions and structural variations on the reaction performance. The second part is devoted to the reaction mechanism with the emphasis on imine ATH and catalyst behaviour under acidic conditions. The review also addresses the asymmetric hydrogenation (AH) of ketones and imines using molecular hydrogen and the application of ATH in pharmaceutical projects. The contributions of our group to each area are included.

## 1. Introduction

**Scheme 1:**
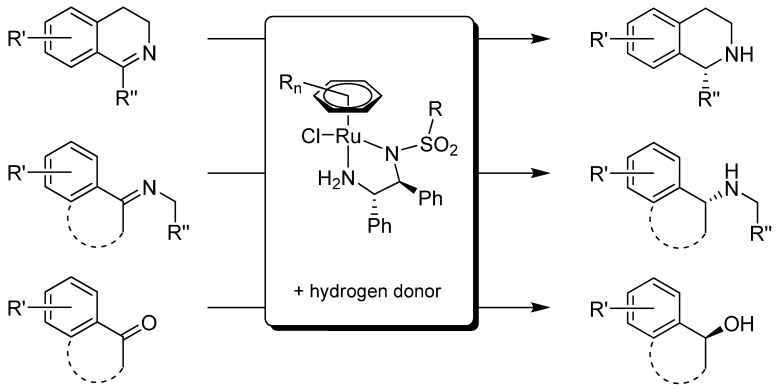
Schematic representation of the ATH of cyclic and acyclic imines and ketones on [RuCl(*η*^6^-arene)(*N*-arylsulfonyl-DPEN)] in the presence of a suitable hydrogen source (isopropanol or HCOOH-triethylamine).

Asymmetric hydrogenation is a well-established and extensively studied method in modern asymmetric synthesis. From 1995–1996, Noyori and co-workers introduced catalytic complexes [RuCl(*η*^6^-arene)(*N*-arylsulfonyl-DPEN)] that were capable of stereoselective reduction of various aromatic ketones [1,2] and imines [3] (Scheme 1). The reduction, commonly referred to as asymmetric transfer hydrogenation (ATH), utilizes isopropanol (only in the case of ketones) or a HCOOH-triethylamine mixture as the hydrogen source. In that way, the use of gaseous hydrogen and pressure reactors can be avoided, which renders the reaction safer and easier to perform.

Since their discovery, these catalysts have experienced extraordinary development in many directions. In our previous review, we described the variety of possible modifications of this particular catalytic system, such as its eco-friendly usage in aqueous media, immobilization on solid supports, incorporation in biomimetic and dendrimeric systems,* etc.*[4].

Despite the number of interesting publications contributing to the development of ATH, little detailed information has been available regarding practical aspects like methods for monitoring the reactions and proper settings for the reaction conditions. Likewise, there are only a few systematic studies on the importance of structural fragments of the catalyst and substrates that form a highly modular system, in which certain combinations are favoured over others. Such fundamental topics are discussed in the first two sections.

The second section is devoted to the reaction mechanism. In our previous work [4], a detailed description of the ATH of ketones was complemented with some general aspects of imine reduction. Therefore, the ATH of imines in acidic environment stands in the focal point of this text, as well as the formation of the Ru-hydride under such conditions.

Finally, the attention is again turned to the practical side of ATH. Since this catalytic system has originally been reported to be operative with a transfer hydrogen donor, most work done in this area so far has followed this arrangement. However, the catalysts can also be used in the asymmetric hydrogenation (AH) of imines and ketones employing gaseous hydrogen. This can be more practical for the introduction of AH in industrial processes in case the facilities are equipped for work with hydrogen gas. Following the AH section, we provide some examples on the industrial application of enantioselective hydrogenation of imines and ketones.

Rather than being an all-embracing monograph, the purpose of this work is to draw attention to the aforementioned topics by presenting appropriate examples, like in the case of our previous review [4].

## 2. Analytical Methods Tailored for ATH

### 2.1. Monitoring of Reaction Kinetics

In order to study a reaction in detail one needs a set of reliable monitoring techniques. The asymmetric transfer hydrogenation of imines can be followed by using several analytical methods. Kinetic hydrogenation experiments are typically carried out in a stirred batch reactor. In the course of the reaction, the samples are manually collected, worked up and analyzed by chromatographic (GC, LC) or spectroscopic (NMR) techniques. A strong advantage of this approach is its robustness and high flexibility. A plethora of different reaction conditions can be examined thanks to virtually unlimited volume of the reaction vessel, possibility of applying different atmospheres, temperature,* etc.* For the chromatographic analysis, there are a number of chromatographic columns to choose from. On the other hand, the laborious sample collection and workup represent the bottleneck of this method that limits the number of points on the kinetic curve. Inaccuracy is introduced by the timing of sample collection (especially when the reaction is fast and the time intervals are short) and the decreasing volume of the reaction mixture with each sample.

These issues can be resolved by non-invasive monitoring of the reaction* in situ*, which can be easily automated. The automation considerably reduces errors introduced by the personnel and enables the collection of a large number of points on the kinetic curve. For instance, Blackmond* et al.* monitored the ATH of 6,7-dimethoxy-1-methyl-3,4-dihydroisoquinoline on [RhCl(*η*^5^-Cp*)(TsDPEN)] (Cp* = pentamethylcyclopentadienyl) by direct acquisition of IR spectra of the reaction mixture [5]. The absorbance values of imine and amine measured at 1069 and 1138 cm^−1^, respectively, were calibrated on the basis of GC measurements, and the dependence of absorbance on concentration was shown to be perfectly linear.

In our kinetic studies, NMR spectroscopy was often utilized for the monitoring of ATH [6]. The reactions can be performed in standard NMR tubes in a volume of 400–800 μL. The ^1^H-NMR spectra are acquired in regular time intervals by using an automated program. Integration of the spectra provides data points that make up a very precise kinetic curve.

### 2.2. Determination of Enantioselectivity

The determination of enantiomeric excess (*ee*) is an integral part of asymmetric catalysis. For the ATH of dihydroisoquinolines (DHIQs), the *ee* of chiral products (tetrahydroisoquinolines, THIQs) is typically determined by using GC-MS [7,8] or liquid chromatography with UV-Vis [9,10], fluorescent [11] or MS [12,13] detection. The instruments are usually equipped with columns with a chiral stationary phase that allows direct separation of enantiomers. The usage of chiral GC columns is limited by lower thermal stability of their stationary phase, and thus only analytes that are volatile enough can be analyzed. On the other hand, the analysis on a chiral LC column often suffers from low peak resolution [10].

**Scheme 2:**
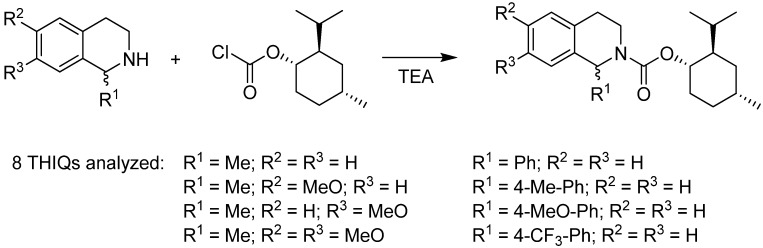
Pre-column derivatization of chiral substituted tetrahydroisoquinolines with (−)-(1*R*)-menthyl chloroformate. The diastereomeric carbamates can be resolved on an achiral GC column and in this way, enantiomeric excess can be determined.

As an alternative, we developed a method for the determination of *ee* on an achiral GC column following pre-column derivatization with cheap (−)-(1*R*)-menthyl chloroformate [14]. In this way, the chiral THIQs form diastereomeric carbamates that are readily resolvable on an ordinary non-polar GC column (Scheme 2). The obtained *ee* values agreed with the ones reported in the literature. The achiral column tolerates higher operational temperatures, and thus it is even possible to analyze less volatile compounds, which are incompatible with temperature-sensitive chiral columns. The method was validated, providing limits of detection and quantification.

Another common way of measuring *ee* is the usage of chiral reagents such as Mosher’s acid (a derivatization reagent giving resolvable diastereomers) [15] or Pirkle’s alcohol (a chiral solvation reagent) [16]. Chiral amines with a high boiling point, such as 6,7-dimethoxy-1-(3',4',5'-trimethoxybenzyl)-1,2,3,4-tetrahydroisoquinoline (a precursor of the skeletal muscle relaxant mivacurium chloride), cannot be analyzed by GC and thus derivatization or solvation reagents can be used effectively. For instance, we analyzed the tetrahydroisoquinoline by NMR employing Pirkle’s alcohol [6,17] and in this way, the aromatic signals of the amine’s enantiomers were fully separated in ^1^H-NMR spectra and the *ee *was determined.

## 3. Parameters Governing the Reaction Performance

### 3.1. Influence of the Reaction Conditions

**Figure 1:**
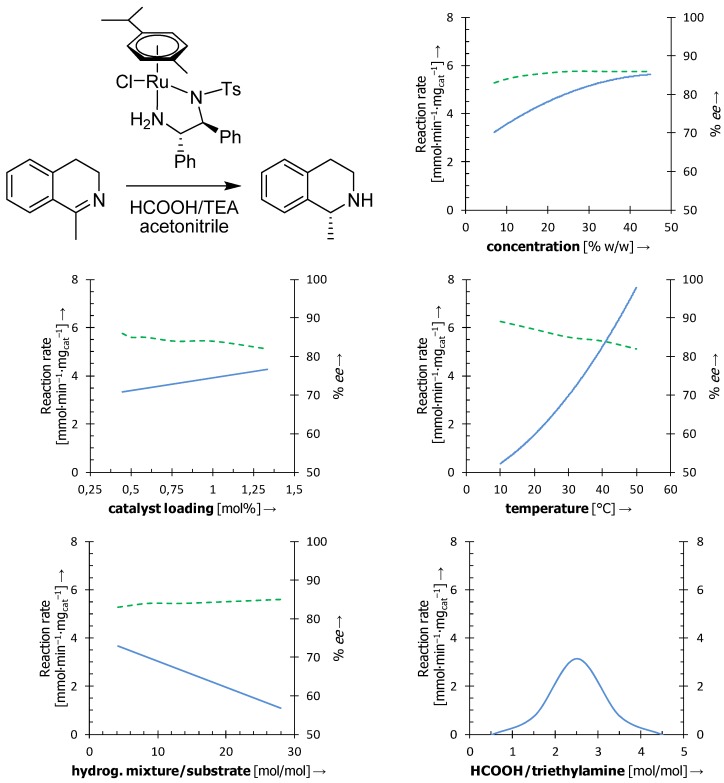
Schematic representation of the influence of individual parameters on the reaction rate (blue solid line) and/or enantioselectivity (green dashed line) of the ATH of 1-methyl-3,4-dihydroisoquinoline on [RuCl(*η*^6^-arene)(TsDPEN)] [19].

In the course of our experiments, we very soon found that the reaction performance is sensitive to proper setup of a number of parameters. The information regarding reaction conditions is very scarce in the literature, which led us to perform extensive screening experiments in order to determine the importance of individual parameters (Figure 1) [18].

As expected, we found that the ATH of dihydroisoquinolines depends on the concentration of the reaction mixture. With increasing concentration, the reaction rate increased until it asymptotically approached a maximum value, where the reaction mixture contained excess of the HCOOH-triethylamine mixture over the solvent. Enantioselectivity remained almost intact. Catalyst loading was the second parameter involved in our screening. Within the tested range (0.44–1.33 mol%), the reaction rate showed a linear increase with increasing catalyst loading, while the enantioselectivity slightly decreased. Another important factor was the temperature of the reaction mixture. The dependence of the reaction rate on temperature displayed exponential growth within 10–50 °C, which conforms to the Arrhenius equation, but the the enantioselectivity was notably decreased. This can be explained by the transition state theory, in which the energetically more demanding pathway (leading to the undesired enantiomer) becomes more accessible at higher temperatures [19].

So far, only general aspects of the reaction conditions have been described. However, the performance of this particular reaction is also affected by the composition of the reaction mixture. For instance, we examined the amount of the HCOOH-triethylamine hydrogenation mixture. Surprisingly, the more hydrogenation mixture we applied, the lower reactivity was observed. This can be caused by the decomposition of the catalyst in the presence of an acid, which protonates the diamine ligand and causes its decoordination. Another explanation might involve the protonated triethylamine forming a hydrogen bond with the sulfonyl group of TsDPEN, thus occupying the active site and decreasing the reaction rate. Enantioselectivity, however, did not change significantly. The optimal molar ratio of the HCOOH-triethylamine mixture was confirmed to be 5:2 as originally disclosed by Noyori* et al.* [3]. The low reaction rates obtained at different ratios,* i.e.*, at excess of the acid or triethylamine, can be explained similarly by decoordination of the diamine, or by “antagonism” between triethylamine and the substrate, respectively. In a similar study focused on the ATH of ketones, the optimal HCOOH-triethylamine ratio was found to be 1:5 [20]. Interestingly, the schematic Gaussian-like curve conspicuously resembles the pH-activity dependence in the ATH of acetophenone on [RhCl(*η*^5^-Cp*)(MsDPEN)] and [IrCl(*η*^5^-Cp*)(MsDPEN)] (MsDPEN = *N*-(methanesulfonyl)-1,2-diphenyl-ethylenediamine) [21]. 

Zhang and co-workers showed that different amines in the hydrogen-donor mixture can affect the enantioselectivity of the ATH of a ketone [22]. In a similar fashion, we carried out the screening of several secondary, tertiary and aromatic amines in the ATH of dihydroisoquinolines. The results were not uniform. In the case of a chiral substrate, (*R*)-1,4-dimethyl-3,4-dihydroisoquinoline, the reaction rate and stereoselectivity were strongly dependent on the selection of the amine [23]. Differences were also observed in the ATH of 1-methyl-3,4-dihydroisoquinoline but the rates and *ee*s did not follow the same trend. The differences among *ee*s were not as pronounced as in the case of the chiral substrate. Eventually, when such screening was performed with 6,7-dimethoxy-1-(3',4',5'-trimethoxybenzyl)-3,4-dihydroisoquinoline, a precursor of mivacurium chloride, only the reaction rates varied while *ee* was not changed in most cases [17]. Therefore, the importance of selection of the base appears to be highly dependent on the studied substrate.

### 3.2. Structural Effects of the Catalyst

In our previous review, we described the modifications of the catalyst directed towards its heterogenization, usage in aqueous media and ionic liquids,* etc.*[4]. This work is focused on the modifications of the catalyst with the aim of increasing the catalytic performance (reaction rate and enantioselectivity) with certain substrates. The Ru^II^ catalyst consists of three main parts that can be modified: the *η*^6^-aromatic ligand, chiral diamine, and chloride anion (Scheme 1).

The *η*^6^-arene is considered responsible for the asymmetric bias of the reaction *via* a CH/*π* interaction between the *π* electrons of the substrate and hydrogen atoms of the arene’s C–H bonds (see Section 4) [24]. Markedly strong dependence of *ee* and catalytic activity on the selection of the ligand was reported in the ATH of acetophenone on [RuCl(*η*^6^-arene)(2-methylamino-1,2-diphenylethanol)] and [RuCl(*η*^6^-arene)(2-amino-1,2-diphenylethanol)], (*η*^6^-arene = benzene, *p*-cymene, mesitylene and hexamethylbenzene) [25]. On the contrary, Yamada and Noyori showed that the *ee* did not change significantly in the ATH of benzaldehyde-1-*d*[26]. Similarly, the differences in reactivity were small except for the case of *η*^6^-hexamethylbenzene, the reactivity of which was an order of magnitude lower than that of the others. This implies that the effects are highly specific for each combination of the substrate and catalyst – some substrates are less prone to the effects introduced by the aromatic ligand.

The chiral diamine, typically being TsDPEN, can be altered in various ways by: (a) functionalization of the aromatic rings (reviewed previously [4]), (b) changing of the sulfonyl substituents, (c) substitution at the amino group, and (d) modifying the spacer length. 

**Figure 2:**
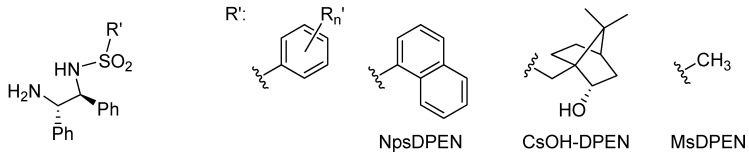
Examples of reported N-aryl and N-alkylsulfonyl diamine ligands.

The sulfonyl group typically features substituents like *p*-tolyl and mesityl [1,2,3]. Modification of this moiety (Figure 2) can lead to a significant change in catalytic activity and enantioselectivity in the ATH of certain substrates. For example, employing the 1-naphthylsulfonyl-1,2-diphenylethylene-1,2-diamine ligand (NpsDPEN) enabled the reduction of 1-phenyl-substituted DHIQs [3,27,28]. Methanesulfonyl-1,2-diphenylethylene-1,2-diamine (MsDPEN) proved very useful in the asymmetric hydrogenation of ketones and imines with molecular hydrogen (*vide infra*) [29,30,31,32]. Very recently we reported a catalyst containing *N*-(borneol-10-sulfonyl)-DPEN (CsOH-DPEN [33]) which was able to catalyze the ATH of both 1-alkyl- and 1-phenyl-DHIQs [34]. This ligand and its reduced analogue *N*-(camphor-10-sulfonyl)-DPEN (CsDPEN) have been previously used in the preparation of several enantiopure ketones *via* ATH [33,34,35,36,37].

**Figure 3:**
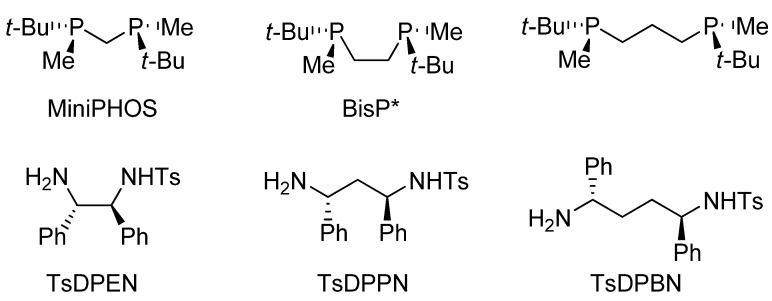
P,P and N,N ligands with spacers of different length.

Changing the length of the spacer (Figure 3) within the diamine ligand leads to a notable change of the bite angle. This is reflected in the stability of the catalytic complex as well as its reactivity and selectivity. For instance, in the case of bidentate phosphine ligands PRR'-(CH_2_)*_n_*-PRR' (*n* = 1–3, R = Me, R' = *t*-Bu), only the bis(phosphino)methane and bis(phosphino)ethane derivatives (MiniPHOS and BisP*) performed with high enantioselectivity in the AH of *Z*-*α*-acetamidocinnamate (99.0 and 99.9% *ee*, respectively), while the propane derivative displayed poor selectivity (14% *ee*) due to undesired flexibility of the ligand [38]. In a similar fashion, we prepared *N*-*p*-toluenesulfonyl-1,3-diphenylpropylene-1,3-diamine (TsDPPN) and *N*-*p*-toluenesulfonyl-1,4-diphenylbutylene-1,4-diamine (TsDPBN). Despite the fact that our DFT calculations indicated possible usability of TsDPPN in the ATH of DHIQs, no catalytic activity was achieved with [RuCl_2_(*η*^6^-*p*-cymene)]_2_. We believe that this is due to the ligand flexibility, which is responsible for its weak coordination to ruthenium. In the case of TsDPBN, molecular modelling even suggested that the formation of a Ru^II^ complex would be rather unlikely, which was confirmed experimentally [39].

**Figure 4:**
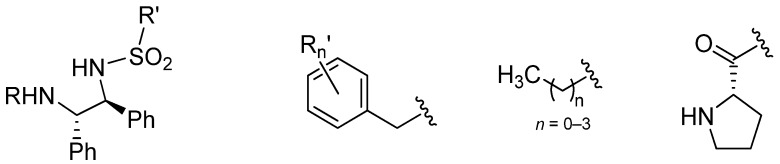
Examples of reported N'-alkylated diamine ligands.

Some examples of substitution of the amino group are known (Figure 4). Initially, the complexes of N'- and N',N'-dialkylated TsDPEN were synthesized mainly for the purposes of mechanistic studies (discussed in Section 4). However, a systematic study on the influence of the N'-alkyl group on the ATH of ketones and imines was performed by Wills and co-workers in 2009 [40]. The screening revealed that [RuCl(*η*^6^-benzene)(*N*'-Me-TsDPEN)] was more active than the original [RuCl(*η*^6^-benzene)(TsDPEN)] in the ATH of both acetophenone and 6,7-dimethoxy-1-methyl-3,4-DHIQ. The alkylated catalyst was further examined on a range of acetophenone derivatives, and an extended series of N'-alkylated TsDPEN-containing catalysts was tested, giving higher *ee*s compared to the non-alkylated complex [41]. In 2011, they introduced a proline group onto the NH_2_ group of TsDPEN *via* an amidic bond. The catalyst was suitable for the ATH of various aromatic ketones in water [42]. Another reported TsDPEN analogue is one that contains a triazole moiety, also applicable in the ATH of ketones [43]. However, although it seemingly belongs to this category, it was reported with Ru_3_(CO)_12_ instead of [RuCl_2_(*η*^6^-arene)]_2_, which implies a different mode of coordination and a different reaction mechanism.

**Figure 5:**
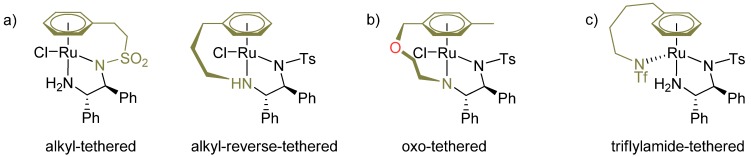
Examples of tethered complexes of Wills and Ikariya.

The ultimate modification of both aromatic ligand and diamine is their interconnection by a chain of several atoms, commonly called tether (Figure 5a). As the first example, Wills and co-workers reported a complex where the *η*^6^-arene and sulfonyl group are connected by a C_2_-tether [44]. In the structure of the original catalysts, the aromatic ligand can rotate freely, which opens up the possibility of formation of a number of different transition states. On the contrary, the arene in the tethered complex keeps a specific orientation. The rigidity of the ligand positively contributes to the asymmetric induction, which was shown on the ATH of a number of aromatic ketones. Subsequently, a reverse-tethered ligand was reported, in which the tether connects the *η*^6^-arene with the amino group [45]. Similarly, the catalyst was tested in the ATH of aromatic ketones and its activity (the catalyst was active at a substrate-to-catalyst ratio S/C = 10,000) was found to be substantially higher compared to the original non-tethered catalysts. The tethered concept has been developed into a set of catalysts [44,45,46,47,48,49,50,51], most of which have been reviewed previously [52]. Recently, Ikariya* et al.* developed a new generation of tethered catalysts by incorporating a heteroatom (oxygen) in the tether (Figure 5b) [53]. The oxo-tethered catalysts were likewise very active and selective in the ATH of a series of ketones, where an impressive S/C ratio of 40,000 was reached. Wills* et al.* showed its applicability in the ATH of a cyclic imine (6,7-dimethoxy-1-methyl-DHIQ) and introduced an alternative synthetic path toward the oxo-tethered Ru^II^ catalyst [54].

The catalysts typically feature a chloride anion coordinated to the ruthenium atom. As shown by Sandoval, Noyori and co-workers [55], the presence of the Ru–Cl bond introduces a number of species described as covalent complex, oligomers, ion pairs and even free ions. The ratio of those species highly depends on the solvent applied. When dissolved in methanol, the complex mostly exists as a mixture of solvent-separated ion pairs and free ions, whereas in dichloromethane almost no ionization occurs. The ionization of the catalyst was promoted by at lower concentrations owing to a higher amount of solvent molecules that facilitate the ionization. In parallel, analogous complex featuring a triflate anion instead of the chloride was examined. This complex displayed much more facile ionization, and thus was more suitable for asymmetric hydrogenation (AH) employing gaseous hydrogen (*vide infra*). Fan* et al.* [31] tested a series of different counteranions (OTf^−^, BF_4_^−^, PF_6_^−^, SbF_6_^−^ and BArF^−^) and showed that they can even influence the enantioselectivity: in the AH of acetophenone *N*-benzylimine, a Ru-triflate complex gave a product with 89% *ee*, while the Ru-BArF^−^ complex afforded 95% *ee*.

In 2008, Ikariya* et al.* introduced a novel concept of tethered catalysts where the NTf^−^ counteranion is connected to the aromatic ligand by a chain of carbon atoms (Figure 5c). The catalysts were especially designed for the asymmetric hydrogenation with gaseous hydrogen because the triflylamide group is deemed to facilitate the activation of molecular hydrogen and thereby the formation of the Ru-hydride species. So far, such complexes of Ru [56], Rh and Ir [57] have been presented in the AH of aromatic ketones. The tether length was found to be very important in relation to the hydrogenation experiments. In all cases, the C_4_ tether gave the highest activity and enantioselectivity.

### 3.3. Structural Effects of the Substrate

The reaction performance is strongly dependent on the substrate-catalyst combination. This fact is already evident from Noyori’s original studies [1,2,3], in which each substrate required different modification of the catalytic complex. However, the complete screening results have not been disclosed and therefore no structure-activity relations can be deduced from the data available.

**Figure 6:**
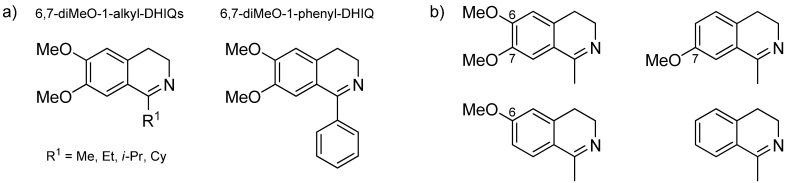
Examples of (**a**) 1-alkyl- and 1-aryl-DHIQs and (**b**) methoxylated derivatives of 1-methyl-DHIQ.

The molecule of DHIQs typically bears an alkyl or aryl substituent in position 1 (Figure 6). The first screening of a series of 6,7-dimethoxy-1-alkyl-DHIQs (alkyl = Me, Et, *i*-Pr, Cy) on related complex [RhCl(*η*^5^-Cp*)(TsDPEN)] was done by Mao and Baker in 1999 [58]. With all substrates, the catalyst displayed high activity (full conversion in 10 min at 0.5% catalyst loading and 20 °C) and good-to-excellent enantioselectivity. However, the trend was not linear (90, 83, 99 and 94% *ee*, respectively), which implies that the relationship of structure and *ee* is not trivial in this case. In 2006, Zhu, Deng* et al.* examined the ATH of three DHIQs (alkyl = Me, Et and *i*-Pr) on [RuCl(*η*^6^-*p*-cymene)(*o*,*o*'-disulfonyl-TsDPEN)] in water containing a surfactant and sodium formate [59]. While the enantioselectivity decreased with increasing length of the substituent (95, 92 and 90% *ee*, respectively), 6,7-dimethoxy-1-methyl-DHIQ outperformed the other two substrates in terms of reactivity.

It is known that the reactivity of 1-alkyl-DHIQs is distinctively higher than that of 1-phenyl-DHIQs. In the original work [3], Noyori* et al.* showed that the ATH of 6,7-dimethoxy-1-phenyl-DHIQ can be catalyzed by [RuCl(*η*^6^-benzene)(NpsDPEN)]. However, the manipulation with this catalyst is complicated due to its very low solubility. Vedejs* et al.* showed that the reaction can be catalyzed by [RuCl(*η*^6^-benzene)(TsDPEN)], the solubility of which is considerably higher [27]. Hydrogenation on [RhCl(*η*^5^-Cp*)(TsDPEN)] proceeded with similar activity but almost no enantioselectivity (3–4% *ee*) [58]. Recently, we showed that [RuCl(*η*^6^-*p*-cymene)(CsOH-DPEN)] can hydrogenate 1-phenyl-DHIQs with performance similar to that of [RuCl(*η*^6^-benzene)(NpsDPEN)] [34]. Zhu, Deng and co-workers demonstrated that the substrate can be activated by converting it into a benzyliminium derivative [59]. The group of Pihko supported the necessity of substrate activation by using Lewis acids (e.g., La(OTf)_3_) [60] coupled with catalyst activation by AgSbF_6_ (see Section 5 for the description of activation of the Ru-Cl bond). Currently, the studies of ATH of 1-aryl-DHIQs are particularly focused on highly active iridium-phosphine complexes, which afford products often with great asymmetry (see e.g., [61]).

Our group performed an investigation into the substitution of the DHIQ molecule with methoxy (MeO) groups in positions 6 and 7 [62]. When hydrogenated on [RuCl(*η*^6^-*p*-cymene)(TsDPEN)] with HCOOH-triethylamine, the substrates [shown in Figure 6(b)] displayed significant differences in reactivity. While the rate of ATH of 7-MeO-1-Me-DHIQ was highest, substrate 6-MeO-1-Me-DHIQ was the least reactive. Out of the remaining substrates (6,7-diMeO-1-Me-DHIQ and 1-Me-DHIQ) lying in between, the double-methoxylated one was more reactive. Therefore, it could be assumed that the increase of the reaction rate is induced by the 7-methoxy substitution to a greater extent than the reaction slowdown caused by the 6-methoxy group. The reactivity of the DHIQs seems to be related to the p*K*_a_ of their conjugate acids.

## 4. Reaction Mechanism

### 4.1. Formation of the Ruthenium-Hydride Species

The mechanism of ATH of the C=X double bond (X = N, O) using the [RuCl(*η*^6^-arene)(*N*-arylsulfonyl-DPEN)] catalytic system can be divided into three fundamental parts—(1) generation of the catalytically active species (hydride) from the chloride ‘precatalyst’, (2) reduction of the double bond with concomitant generation of asymmetry, and (3) regeneration of the catalyst. 

In the case of ketones, all mentioned steps have been extensively studied and we discussed them in our previous review [4]. However, very recently Dub and Ikariya reported a detailed DFT study [63] which shows that the formation of concerted six-membered pericyclic transition states (through which the ATH of ketones was deemed to operate) solely conformed to gas-phase calculations. They proposed that in solution, the hydrogenation occurs *via* a two-step pathway in which the hydride is transferred first (generating the asymmetry), followed by a proton transfer from the solvent molecule or TsDPEN.

In this work, emphasis is placed on detailed description of the third step,* i.e.*, regeneration of the Ru-hydride species in the presence of the HCOOH/triethylamine mixture. The Ru–Cl bond dissociation (which belongs to step 1 of the mechanistic pathway) is discussed in Section 5 and hydrogenation (step 2) is addressed within the description of the ATH of individual classes of substrates (*vide infra*). We would also like to extend our focus on the acidic environment (*i.e.*, formic acid with triethylamine) of the hydrogenation of C=N bond. At this point it is worth mentioning that the spectrum of substrates/bonds which can be hydrogenated using the aforementioned catalysts was recently extended to activated olefins, which is described in Section 4.3.

Precise mechanism of the formation of catalytically active species (ruthenium hydride) in acidic environment has not yet been fully and consistently described. It is generally assumed that the formation of the Ru-hydride is preceded by attraction of the formate anion into the ‘cavity’ of the 16e^−^ complex (or Ru-solvate under acidic conditions) *via* non-covalent interactions leading to the creation of the Ru-formate species [64]. This species is difficult to isolate and readily gives the Ru-hydride through decarboxylation, which is reversible: under the atmosphere of pressurized CO_2_, the Ru-formate reappears. We even observed notable inhibitory effect of CO_2_ (*p*(CO_2_) = 1 atm) on the reaction rate of imine ATH [18], the reason of which is believed to be the formation of the Ru-formate.

**Scheme 3:**
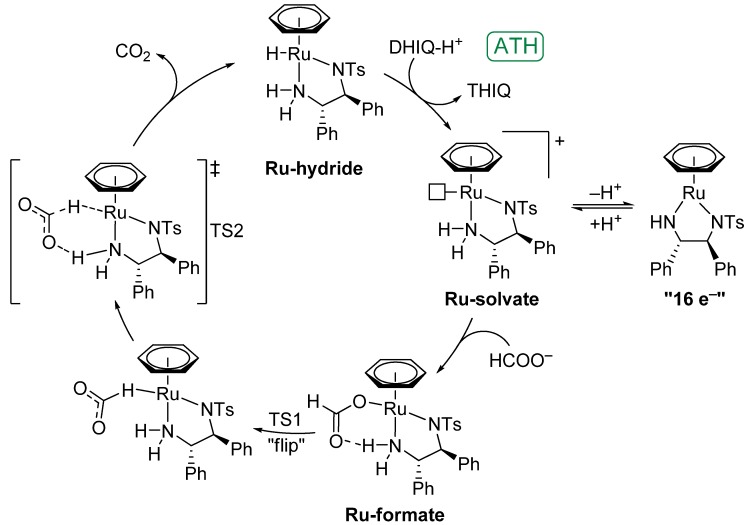
Proposed mechanism of Ru-hydride formation from the Ru-solvate (*i.e.*, protonated 16e^−^ complex) and formate ion.

The HCOO^−^ ion is bound *via* a Ru–O bond in the Ru-formate complex and is further stabilized by a C=O∙∙∙H–N hydrogen bond with the TsDPEN ligand. In order to form a Ru-hydride from the Ru-formate, the anion must reorient so that its hydrogen atom points toward ruthenium, which is denoted as a “flip” in Scheme 3. Since the coordination sphere of the Ru-formate is saturated, it is quite likely that one of the ligands should temporarily decoordinate for the flip to occur. Koike and Ikariya suggested three ways of decoordination [64]: *η*^6^ to *η*^4^ slippage, formation of an ion pair by dissociation of the formate, or partial decoordination of TsDPEN. The ring slippage has been proposed in the case of analogous complexes containing 2,2'-bipyridine ligand, which also serve as transfer hydrogenation catalysts for the synthesis of optically enriched alcohols [65,66].

Therefore, the Ru-hydride formation probably requires two consecutive transition states. In the first one (TS1), the HCOO^−^ ion changes its orientation (“flip”) – a reversed transformation was calculated by Chen* et al.* for the process of CO_2_ reduction on Ir^III^ complexes [67]. Sadler* et al.* [68] computed the hydride formation from [Ru(HCOO)(*η*^6^-benzene)(MsEN)] (MsEN = *N*-methanesulfonyl-ethylene-1,2-diamine) but in that case, no decoordination was considered. In the second transition state (TS2), decarboxylation occurs to afford the Ru-hydride and CO_2_.

**Figure 7:**
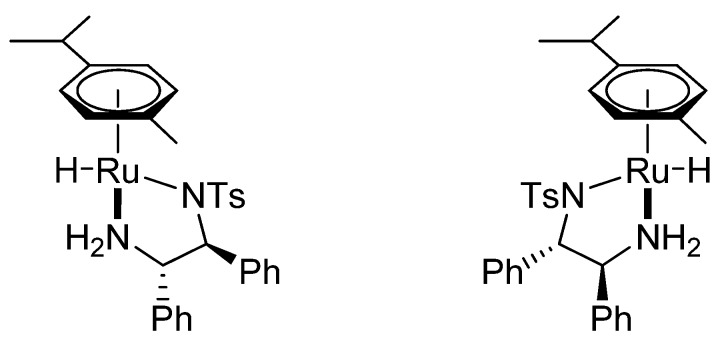
Two diastereomers of [RuH(*η*^6^-*p*-cymene)(*S*,*S*)-TsDPEN]. The structures differ by configuration on the Ru atom, while both feature the (*S*,*S*)-TsDPEN ligand.

The overall description of the mechanism is further complicated. Several independent studies have suggested that two stereoisomers of the Ru-hydride (Figure 7) can arise from one enantiomer of the Ru-chloride. Two sets of signals corresponding to the hydride species were observed by us in the ^1^H- NMR spectra of a mixture of [RuCl(*η*^6^-*p*-cymene)(TsDPEN)], triethylamine and HCOOH [23]. Wills* et al.* described similar behaviour with his tethered catalysts and showed that one of the isomers is probably much less active in the ATH of acetophenone than the other [49], which was also demonstrated by Rauchfuss* et al.* on [Ir(*η*^5^-Cp*)(TsDPEN)]^+^[69]. We speculate that this diastereoselective transformation of the Ru-chloride to two Ru-hydride species is caused by the ruthenium complex itself (which serves as a chiral selector) since no other chiral component is present in the mixture. On the contrary, only one Ru-hydride species was observed with achiral complex [RuCl(*η*^6^-*p*-cymene)(TsEN)] (TsEN = *N*-tosyl-ethylene-1,2-diamine) [64], which further (yet indirectly) supports the hypothesis.

It is rather unclear why the diastereomers are only observed upon Ru-hydride formation and not during the synthesis of the Ru-chloride precatalyst from [RuCl_2_(*η*^6^-*p*-cymene)]_2_ and TsDPEN. If the minor isomer is formed, it can possibly be separated by crystallization of the product. In the case of some other catalysts, two sets of signals pertaining to the Ru-chloride species have been observed. For instance, Wills* et al.* described the presence of an impurity in the NMR spectra of one of their complexes [70]. Interestingly, however, when switching from CDCl_3_ to CD_3_NO_2_, the impurity was no longer observed.

### 4.2. Mechanism of ATH of Imines and Catalyst Behaviour under Acidic Conditions

Generally speaking, the asymmetric transfer hydrogenation of C=N and C=O bonds bears many divergences and only few similarities. The original hypothesis [71] claiming that nearly the same mechanistic pathway applies for the hydrogenation of both ketones and imines seems to be refuted by experimental and computational evidence (*vide infra*). What appears to be the same is the origin of enantioselectivity which lies in the attractive CH/π interaction of the *η*^6^-arene ligand with the aromatic part of the substrate. The diamine ligand builds the cavity of the catalyst with a geometry permitting the stabilization *via* the CH/π interaction only for a limited number of substrate orientations. These effects altogether result in a split of the ‘racemic’ reaction coordinate into two separate pathways. In one of the pathways, the substrate orientation allows the formation of a CH/π interaction that stabilizes the transition state (which, in that case, is called a favourable transition state). In the other route, the substrate orientation does not permit the CH/π attraction, and therefore the transition state has a higher energy (disfavourable transition state).

As we mentioned before, there is evidence that the C=O reduction pathway does not operate in the case of imines. The results published by Bäckvall* et al.* [72] show that the imine substrate needs to undergo acidic activation by Brønstedt (e.g., HBF_4_, CH_3_COOH, CF_3_COOH,* etc.*) or Lewis acid (scandium (III) triflate). When no acid was present in the reaction mixture (which consisted of Ru-hydride, imine and dichloromethane), the ruthenium hydride did not react with the substrate, *ergo*the reaction did not proceed (no product was detected even after 31 hours). The usage of lanthanide and bismuth (III) triflates as Lewis acids for substrate activation was also examined by Pihko* et al.* [60] (mainly because of their water solubility and previously reported positive accelerative effects on reactions involving imines).

Mechanistic investigations carried out by Wills and co-workers indicate that it is highly unlikely for the protonated nitrogen of the C=N bond group to interact directly with the amino group of the diamine [51]. The main reason is that alkylation of the amino group with sterically demanding fragments did not lower the reaction rate as much as expected—in fact, the reaction proceeded even with a double-alkylated amino group. This observation represents striking difference from ketones, the reduction of which was strongly slowed down by the bulky alkyl chain on the amino group, and no product was observed when a catalyst with N',N'-alkylated amino group was used. In addition, the only orientation of the substrate permitting the essential CH/π interaction (and not leading to the opposite isomer than the one found in the reaction mixture) is that where the imino group aims towards the tosyl fragment [40].

We unified all of the above-mentioned findings and examined the reaction mechanism *via* means of computational chemistry. Our first paper on this topic was devoted solely to a cyclic imine (1-methyl-DHIQ) and its interaction with catalyst [RuCl(*η*^6^-*p*-cymene)(TsDPEN)] [73]. Unlike highly symmetrical aromatic ligands (benzene, mesitylene or hexamethylbenzene), *p*-cymene offers four different sites for the CH/π interaction; a comparison of transition states which employed different sites showed that they differed in their free energy (yet their single-point energy values were quite close). The stabilizing interaction (a hydrogen bond) between the protonated substrate and oxygen within the sulfonyl group proved to be very important for the minimization of the transition state energy. 

Following the initial computational study, we extended our scope to acyclic imines like acetophenone *N*-benzylimine (ACPBI), which differ from their cyclic analogues in some aspects [74]. The most apparent difference is that the hydrogenation of ACPBI exhibits relatively low *ee* and leads to the prevalence of a product with the same configuration as the diamine ligand (which is not the case of cyclic DHIQs or *β*-carbolines, which always have an opposite configuration). The explanation of this phenomenon partly lies in the fact that, because of its exocyclic C=N bond, ACPBI inherently exists as a mixture of two geometrical isomers that can undergo acid-catalyzed interconversion. Strong structural divergence of both geometrical isomers causes that each of them interacts with the catalyst in a different manner. Our calculations showed that while the *E* isomer is reduced with a very low selectivity to give an (*R*)-amine (and therefore lower enantiomeric excess), the reduction of the *Z* isomer leads to the delivery of an (*S*)-configured product with notably high enantioselectivity. The computations also confirmed that ACPBI is a very flexible molecule contrary to the rigid DHIQs, and allows the formation of multiple CH/π or NH/π interactions.

The acidic environment is associated not only with the protonation of the imine substrate, but also of the base accompanying formic acid (usually triethylamine). In Section 3.1 we described our finding that the selection of a base can affect the reaction rate and stereoselectivity [23]. Studying the molecular system by high-resolution mass spectrometry revealed that the base forms associates with the catalytic complex. Further investigations pointed toward the fact that the protonated base can be hydrogen-bound to the oxygen atoms of the sulfonyl group. Its influence on the reaction rate can thus be explained by competition with the substrate for the sulfonyl group. Stereoselectivity is a question of distribution of favourable and disfavourable transition states [19]. Hence, the presence of the base at the complex *during* the transition states alters their energy distribution,* i.e.* the reaction stereoselectivity.

### 4.3. ATH of Activated Olefins

Study by Xue* et al.* demonstrated that ruthenium diamine complexes (and even their dimeric precursors) are capable of hydrogenation of activated olefins [75]. *α*,*β*-unsaturated compounds were activated *via* electron-withdrawing substituents like cyano or nitro groups in *α* and *β* positions, which led to desired polarization of the C=C bond. In the case of *α*,*α*-dicyanoolefins, even high enantioselectivity was achieved (up to 89% *ee*). The usage of formyl-deuterated formic acid (DCOOH) allowed getting better insight into the mechanistic effects connected with this phenomenon. All deuterium atoms were found to be at the *β*-position of the substrate, which suggests that the formyl hydrogen atom (deuterium) is transferred from ruthenium to the electron-withdrawing *β*-carbon. Nevertheless, no deuterium was detected at the *α*-position even with HCOOD or DCOOD, which may be a result of a rapid H-D exchange at the acidic *α*-H position. To sum up, we can conclude that polarization of the C=C bond of olefins can promote their asymmetric hydrogenation on the catalytic systems described in this work.

## 5. Asymmetric Hydrogenation using Gaseous Hydrogen

Unlike the asymmetric hydrogenation of olefins, which is typically performed with gaseous hydrogen and catalysts containing chiral phosphine ligands [76,77,78], the enantioselective reduction carried out on Noyori’s [RuCl(*η*^6^-arene)(*N*-arylsulfonyl-DPEN)] complexes was initially described in a transfer arrangement. In this way, it is possible to avoid the use of molecular hydrogen, which can represent a notable advantage in conditions where its use is not desired. On the other hand, the residues of hydrogen donors (acetone, isopropanol, triethylamine,* etc.*) remain in the reaction mixture and must be separated from the resulting product of hydrogenation. In order to achieve this, their evaporation appears to be one of the simplest ways, but this may represent a problem if the product is thermally degradable or volatile. Moreover, the removal of those compounds presents an additional unit operation, which is not the case of hydrogenation directly employing gaseous hydrogen. 

The first reaction conditions for the asymmetric hydrogenation of ketones were disclosed by Noyori and co-workers [79]. They found that the reaction mechanism of ruthenium-hydride formation under such conditions requires ionic dissociation of the Ru–Cl bond, the extent of which is strongly dependent on the solvent used [80]. The dissociation was substantially enhanced by replacement of the chloride with triflate because in that case, the ruthenium solvate was formed more readily. Subsequent DFT studies carried out by Fang* et al.* suggested that the triflate anion promotes the formation of the Ru-hydride from the 16e^−^ complex and gaseous H_2_ [81]. 

The catalytic system based on the Ru-triflate was first used for the hydrogenation of 4-chromanone in dry methanol, where an S/C as high as 7000 was reached (100% yield and 96% *ee* at 100 atm and 60 °C during 15 h). Similarly, the [RuX(*η*^6^-*p*-cymene)(TsDPEN)] catalyst (X = Cl or OTf) was employed in the hydrogenation of *α*-chloro aromatic ketones where the triflate complex again proved more efficient than its chloride analogue [82]. Related catalyst [Ir(OTf)(*η*^5^-Cp*)(MsDPEN)] was developed by Ohkuma* et al.* [83] for the AH of *α*-hydroxy aromatic ketones. As shown below, the MsDPEN ligand (in ATH a rare example of N-alkylsulfonyl-DPEN) has become fairly popular in AH employing gaseous hydrogen.

Complementing the original class of complexes, Wills* et al.* reported the AH of ketones and chemoselective reduction of aldehydes on their tethered catalysts [70]. Interestingly, the catalysts did not require modification to Ru-triflate but the “standard” Ru-chloride complexes were used. In a similar manner, Ikariya* et al.* showed that his oxo-tethered catalyst is applicable in the AH of various ketones [53]. With most substrates, its 16e^−^ derivative was notably more reactive than the Ru-chloride, which is understandable since the chloride species must first give the 16e^−^ complex in order to react with the substrate. From the same group, also the triflylamide-tethered catalysts arised [56,57], which are addressed in the end of Section 3.2. In the ATH of acetophenone, Ru^II^ complexes [56] outperformed their Rh^I^ and Ir^I^ analogues [57] both in reactivity and enantioselectivity.

**Figure 8:**
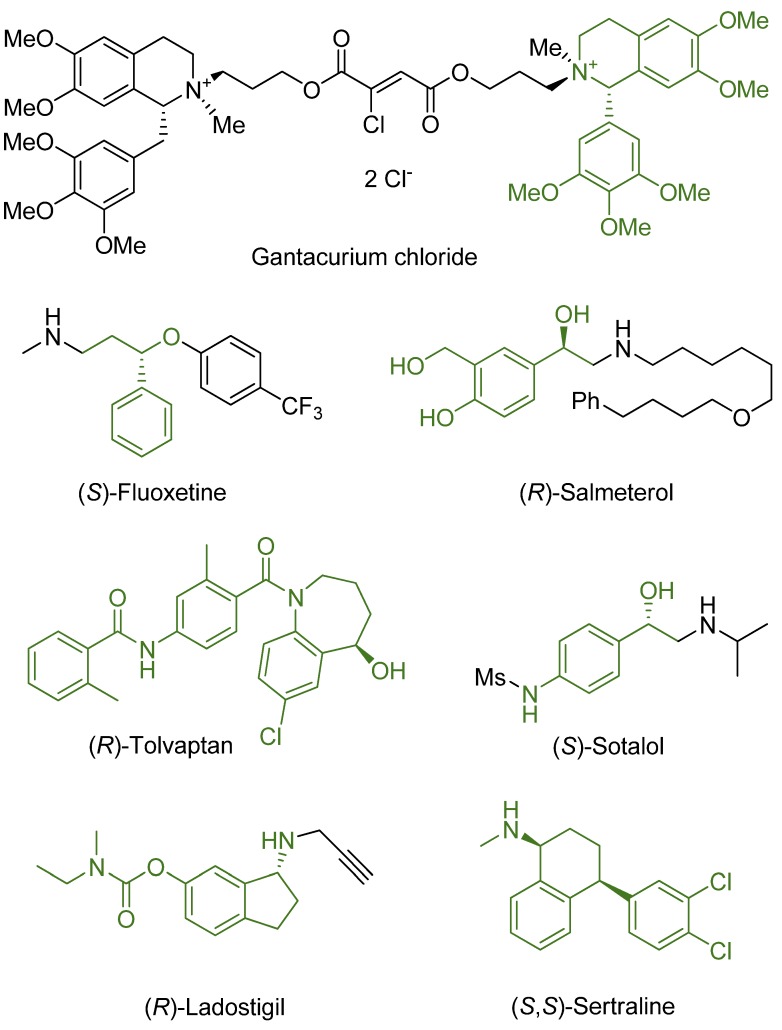
Examples of pharmaceutical products synthesized via ATH or AH using [RuCl(*η*^6^-arene)(*N*-arylsulfonyl-DPEN)] (the chiral building blocks obtained by these reactions are shown in green).

Several alternatives for the AH of imines with molecular hydrogen have also been reported. Xiao and co-workers devised a workaround by adding AgSbF_6_ to the reaction mixture containing [RhCl(*η*^5^-Cp*)(MsDPEN)] [84]. In this way, a rhodium solvate bearing the SbF_6_^−^ counteranion was formed as a product of ionization of the Rh–Cl bond (thereby facilitating the hydride formation). Interestingly, no addition of acid was necessary under given conditions and still the catalyst was more active than its Ru analogue. In a similar fashion, AgSbF_6_ was used for the asymmetric hydrogenation of *N*-(1-phenylethylidene)benzylamine derivatives (acyclic imines) where it increased the enantioselectivity and activity [30]. The group of Fan [31] tested the AH of acyclic imines on [RuX(*η*^6^-*p*-cymene)(MsDPEN)] (X = OTf^−^, BF_4_^−^, SbF_6_^−^ and BArF^−^) and observed decomposition of the substrate to benzylamine, which deactivated the catalyst. The amine was therefore protected by the addition of (Boc)_2_O to the reaction mixture. The best performance was obtained with the catalyst containing the BArF^−^ counteranion. Later, the substrate scope was notably extended and the method was used for the synthesis of sertraline (Figure 8) [85]. A similar study was done with a series of 2-aryl-1-pyrroline derivatives (*i.e.*, cyclic imines) as substrates [86].

## 6. Examples of Practical Usage of ATH

Naturally, the most tangible outcomes of basic research are those that find practical application. Asymmetric (transfer) hydrogenation is not an exception as it has been used in a number of multi-step synthetic pathways. In this section we wish to present several examples to show the wide range of products affordable *via* the ATH of prochiral ketones and imines. The strongest demand for enantiopure compounds comes from the pharmaceutical industry since in most cases, only single enantiomers of active pharmaceutical ingredients (APIs) are allowed to be used in drugs. The ATH products are therefore mostly APIs or their precursors. Selected examples are shown in Figure 8.

The first example was given by the group of Noyori in 1997, soon after the very introduction of their catalytic system: good to excellent enantioselectivity was achieved in the synthesis of precursors of drugs MK-0417 (a carbonic anhydrase inhibitor useful in the treatment of glaucoma) and L-699,392 (an antagonist of LTD_4_) [71]. The group of Samano and Boros followed Noyori and prepared a chiral precursor of the muscle relaxant Gantacurium chloride in 99% *ee*[28]. A substituted 1-phenyl-DHIQ served as the starting material and therefore catalyst [RuCl(*η*^6^-benzene)(NpsDPEN)] was used. In the same fashion, the group continued in the development of other muscle relaxants [87,88]. In 2005, Wang* et al.* prepared a polystyrene-supported TsDPEN, which was used for the preparation of a precursor of (*S*)-fluoxetin with 97% *ee*[89]. Owing to the heterogenization of the catalyst, its reuse was enabled, however, in each subsequent run, the catalytic activity decreased. Li and co-workers immobilized TsDPEN on poly(ethylene)glycol (so-called PEGBsDPEN) and employed it in the synthesis of a precursor of (*R*,*R*)-salmeterol by ATH in PEG-water mixture with [RhCl_2_(*η*^5^-Cp*)]_2_ [90]. While maintaining the *ee* within 97–98%, five-fold recycling was possible but erosion of the activity of the catalyst was apparent. The ligand was used with a similar result in the synthesis of a precursor of (*R,R*)-formoterol [91]. The group of Li applied non-immobilized complexes of CsDPEN in the preparation of tolvaptan [33], clorprenaline and sotalol [36], and ladostigil [37].

Zhang* et al.* used classical [RuCl(*η*^6^-*p*-cymene)(TsDPEN)] in the synthesis of *N*-propylpantolactam (a precursor of *α*-hydroxy-*γ*-amino acids and *α*-hydroxy-*γ*-lactams) [22]. They expanded the spectrum of bases usable in the HCOOH-triethylamine hydrogenation mixture by testing various amines, thus reaching higher *ee*. Furthermore, they performed the synthesis on a big scale and obtained several kilograms of the chiral product. Fan and co-workers utilized the AH by gaseous hydrogen on [Ru(BArF)(*η*^6^-*p*-cymene)(MsDPEN)] for the preparation of enantiopure sertraline (> 99% *ee*) [85].

**Scheme 4:**
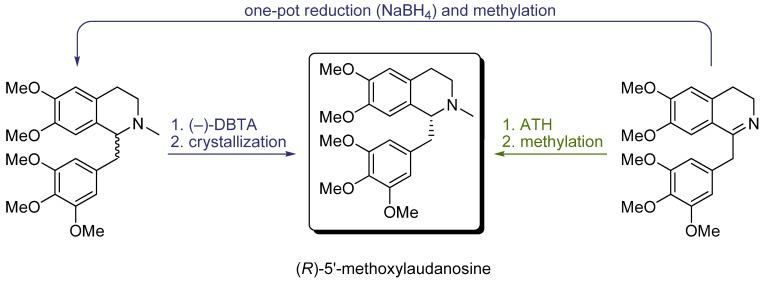
Two synthetic pathways giving (*R*)-5'-methoxylaudanosine.

In our group, we focused on the synthesis of (*R*)-5'-methoxylaudanosine (an enantiopure precursor of mivacurium chloride, gantacurium chloride and other neuromuscular blockers) from 6,7-dimethoxy-1-(3',4',5'-trimethoxybenzyl)-3,4-dihydroisoquinoline (Scheme 4) [17]. The first route to (*R*)-5'-methoxylaudanosine consisted in one-pot reduction and *N*-methylation of the DHIQ, giving racemic 5'-methoxylaudanosine, which was optically enriched by diastereomeric crystallization with (−)-2,3-dibenzoyl-L-tartaric acid. This method is typically used in the industry, however, no detailed reaction conditions were known. The second alternative was the ATH of the dihydroisoquinoline on [RuCl(*η*^6^-*p*-cymene)(TsDPEN)] followed by *N*-methylation. Based on our experience with this ATH system (see Section 3), we optimized the reaction conditions in order to maximize the reaction rate, yield and selectivity. This option gave the target product in considerably higher yield and atom economy. In all cases, the enantioselectivity was very high but further purification was necessary, which was achieved by final diastereomeric resolution.

## 7. Conclusions

The asymmetric reductions of ketones and imines on Noyori-Ikariya Ru^II^ half-sandwich complexes have undergone tremendous development during the almost two decades of their existence. Since the publication of the pioneering experiments, the catalytic system has been adapted to suit numerous substrates and reaction conditions. In this review (which follows our previous paper in *Molecules* on this topic [4]), we have demonstrated a number of such attributes from various viewpoints. The examples together show the way towards practical use of the catalysts in the synthesis of optically enriched products, especially pharmaceuticals. We expect that future development will continue in the design of new catalytic systems and improvement of the existing ones, which will be applicable in the AH and ATH of prochiral substrates.
